# Expression and accumulation of the two-domain odorant-binding protein AaegOBP45 in the ovaries of blood-fed *Aedes aegypti*

**DOI:** 10.1186/1756-3305-6-364

**Published:** 2013-12-24

**Authors:** André Luis Costa-da-Silva, Bianca B Kojin, Osvaldo Marinotti, Anthony A James, Margareth Lara Capurro

**Affiliations:** 1 Departamento de Parasitologia, Laboratório de Mosquitos Geneticamente Modificados, Instituto de Ciências Biomédicas, Universidade de São Paulo, São Paulo, SP 05508-000, Brazil; 2 Instituto Nacional de Ciência e Tecnologia em Entomologia Molecular, INCT-EM, Rio de Janeiro, Brazil; 3 Department of Molecular Biology and Biochemistry, 2305 McGaugh Hall, University of California, Irvine, CA 92697-3900, USA; 4 Department of Microbiology and Molecular Genetics, University of California, Irvine, CA 92697-4025, USA

**Keywords:** *Aedes aegypti*, Odorant-binding protein, OBP, Ovaries, Atypical, Two-domain

## Abstract

**Background:**

*Aedes aegypti* mosquitoes are the main vectors of dengue viruses. Despite global efforts to reduce the prevalence of dengue using integrated vector management strategies, innovative alternatives are necessary to help prevent virus transmission. Detailed characterizations of *Ae. aegypti* genes and their products provide information about the biology of mosquitoes and may serve as foundations for the design of new vector control methods.

**Findings:**

We studied the *Ae. aegypti* gene, AAEL010714, that encodes a two-domain odorant-binding protein, AaegOBP45. The predicted gene structure and sequence were validated, although single nucleotide polymorphisms were observed. Transcriptional and translational products accumulate in the ovaries of blood fed females and are not detected or are at low abundance in other tissues.

**Conclusions:**

We validated the *Ae. aegypti* AAEL010714 gene sequence and characterized the expression profile of a two-domain OBP expressed in ovaries. We propose that AaegOBP45 function as a component of the mosquito eggshell.

## Findings

### Background

*Aedes aegypti* is the main vector of dengue, viruses that causes 390 million infections per year worldwide [1]. Novel methods for the control of this vector to impair dengue transmission are needed urgently. Mosquito genomics and post-genomic research provide new opportunities to explore the biology of mosquitoes and support innovative strategies in vector control [2].

Odorant-binding proteins (OBPs) are present in a wide range of insect species and experimental evidence shows their involvement in chemoreception [3-6]. However, additional functions have been proposed for these proteins [7-9]. Subgroups of OBPs have been defined according to their amino acid sequences. Atypical OBPs were identified first in *Anopheles gambiae*[10], and subsequently in *Ae. albopictus*[8], *Ae. aegypti* and *Culex quinquefasciatus*[11]. Atypical OBPs contain two amino acid sequence domains similar in sequence to Classic OBPs [12]. Although it was proposed that atypical OBPs are unique to mosquito species [8,10,11,13,14], structural similarities support their inclusion in a family of proteins called Dimer OBPs, described previously in *Drosophila*[15,16]*.* Accordingly, atypical OBPs have been renamed as two-domain OBP proteins [11]. *Aedes aegypti* OBP45 (AaegOBP45) is encoded by the gene AAEL010714 and belongs to the *matype4* protein cluster [11]. AAEL010714 is up-regulated by blood feeding, attaining a maximum transcript accumulation at 48 hours post blood meal (hPBM), a pattern similar to other *Ae. aegypti* two-domain OBP genes [17]. Moreover, four OBPs from *An. gambiae* (OBP11, OBP1, OB44 and OBP13) have a high abundance of their corresponding transcripts also at 48 hPBM and were characterized as mosquito eggshell components [18]. Here we describe the accumulation and distribution of a two-domain odorant-binding protein, AaegOBP45, and its corresponding mRNA.

## Methods

### Mosquitoes

*Aedes aegypti* (Higgs white-eye strain) larvae, pupae and adults were reared using standard laboratory procedures [19].

### DNA and RNA extractions and first-strand cDNA synthesis

Genomic DNA extraction was carried out on a pool of 5 mosquitoes using DNeasy Blood & Tissue kit (Qiagen). Whole-body and tissue total RNA preparations were made using TRIZOL (Invitrogen). Samples were extracted from 4^th^ instar larvae, early pupae (0–4 h post-pupation), late pupae (24 h post-pupation), adult males and females (1-day old) and 5-day old females (sugar-fed or 24, 48, 60, 72, 84 and 96 hPBM). Additional samples were derived from 48 hPBM females tissues (fat bodies, ovaries, midguts and heads) dissected in 1x phosphate-buffered saline (PBS). Samples were resuspended in DEPC-treated water and stored at −70°C until required. Total RNA (2.2 μg) was treated with DNaseI (Invitrogen). Approximately 0.2 μg of each treated RNA sample was used as the template for gene amplification with β-actin oligonucleotide primers to confirm the absence of genomic DNA contamination. First-strand cDNA synthesis was done using 2.0 μg of treated total RNA (except from head samples, for which 0.7 μg was used). Oligo(dT)12-18 primer or gene-specific reverse primers and SuperScript III (Invitrogen) were used for RT-PCR.

### Amplification procedures

Oligonucleotide primers designed for AAEL010714 genomic amplification of the corresponding coding region as well as primers for RT-PCR and qRT-PCR experiments, were designed using Primer3 software (http://bioinfo.ut.ee/primer3-0.4.0/) (Additional file 1). The internal forward primer, In-F, was used in combination with the reverse primer to generate a 150 base-pairs (bp) fragment for tissue-specific and qRT-PCR analysis of AAEL010714 mRNA accumulation.

RT-PCRs were performed in final reaction volumes of 25 μL, containing 2.0 mM Mg^++^, 20.0 mM Tris–HCl (pH 8,4), 50 mM KCl, 0.2 μM of each respective primer, 0.2 mM each dNTP, 2.5 U of Taq DNA polymerase (Invitrogen) and 2 μL of cDNA. The thermocycler program was configured for an initial denaturation step at 94°C for 2 m, followed by 30 cycles (except for 18S ribosomal RNA – 20 cycles) at 94°C for 1 m, 60°C for 30 s and 72°C for 1 m, and a final elongation step at 72°C for 7 m. The annealing temperature was 58°C for the AAEL010714-RA transcript template.

qRT-PCR assay was performed in Mastercycler Realplex 2 thermocycler (Eppendorf) with ABSOLUTE™ QPCR SYBR® Green Mix (ABgene). Amplification conditions were 95°C for 15 m, followed by 45 cycles of 94°C for 30 s, 60°C for 30 s and 72°C for 30 s. Serial dilutions of target plasmids containing the 150 bp cloned cDNA fragments in concentrations ranging from 10^-9^ g to 10^-16^ g were used to generate standard curves to obtain the number of transcript copies. Each pool of 10 vitellogenic female samples (36, 48, 60, 72, 84, 96 hPBM) was analyzed in triplicate and the assay was repeated three times with independent biological samples, except for 84 hPBM, which was measured in duplicate. The copy number of β-actin transcripts was used to normalize variation in total cDNA concentration as an endogenous control (Additional file 2) [20,21]. Statistical analyses were performed using GraphPad Prism® (version 5.00) for Windows (GraphPad Software, San Diego, CA, USA). One-way analyses of variance following Tukey’s Multiple Comparison test were used to estimate statistical significance.

### Amplification and cloning procedures for isolation of the AAEL010714 putative gene promoter region and transcription factor binding site prediction

A 2.5 kilobase (kb) region at the 5′-end of the putative translation initiation site of the AAEL010714 gene was identified based on the nucleotide sequence of Supercontig 1.500 (obtained at Vectorbase.org). A primer pair was designed using Primer3 software and included restriction endonuclease cleavage sites for *Bam*HI (Additional file 1). PCR was performed using genomic DNA as the template and TAQ platinum DNA polymerase (Invitrogen) with the amplification cycles 2 m at 94°C, followed by 35 cycles of 1 m at 94°C, 30 s at 60°C, 2 m at 72°C and a final step of 10 m at 72°C. Amplified products were cloned and sequenced using M13For and M13Rev primers and three internal gene promoter primers (Additional file 1). The consensus DNA sequence was submitted to transcription factor binding sites prediction analyses using the JASPAR database [22] based on *Drosophila melanogaster* JASPAR matrix models with a defined 95% profile score threshold. Manual searches were performed for other motifs.

### Alignments, commercial synthesis of peptide and antisera production

Nucleotide and protein alignments were performed using BioEdit software [23]. The peptide GNQFSSSDIDGL was used to generate an AaegOBP45 antiserum. The peptide sequence was subjected to a Blastp search against the NCBI database to ensure that it represents a unique sequence (<10% overlap). Specifically, the peptide sequence was selected to prevent cross-reactions of the antiserum with a related protein, AaegOBP44, encoded by AAEL01718. Polyclonal antiserum to AaegOBP45 was generated in rabbits using a standard protocol (Proteimax Biotechnology Ltda., Cotia, São Paulo-Brazil).

### Immunoblot analysis

Proteins extracted from ovaries dissected at 24, 48 and 72 hPBM (50 μg for each time point) were fractionated by SDS-PAGE on a 12% polyacrylamide gel. Duplicate gels either were stained with 0.1% Coomassie Blue G-250 solution or electroblotted onto Trans**-**Blot transfer medium**-**supported nitrocellulose membrane (Bio-Rad). Membrane blocking, antisera incubations and detection procedures were performed following instructions described in the ECL Plus product booklet and detection of immune reacted moieties was carried out using ECL Plus Western blotting reagent (GE Healthcare). Primary antiserum was used at a dilution of 1:500 and HRP-conjugated secondary antibodies at 1:2500.

## Results

### AAEL01714 gene sequence

Nucleotide polymorphisms are anticipated between mosquito strains [24] and therefore 3,716 bp of genomic DNA corresponding to the AAEL010714 coding sequence and putative promoter from the Higgs strain were sequenced and compared with the Liverpool reference genome (Vectorbase.org). The genomic sequences showed 99.8% identity and nucleotide polymorphisms were observed only in putative promoter regions.

The putative 5′-end regulatory region of AAEL010714 contains a canonical TATA box and an arthropod initiator motif (*Inr*). Additionally, potential binding sites for transcription factors involved in the hierarchical control of genes induced by ecdysone during reproduction were identified [20] (Additional file 3).

AAEL010714 has a paralogue, AAEL010718, with 95% identity in the coding sequences. Although both transcripts are annotated in Vectorbase, RT-PCR experiments using primer pairs F and RT-R or IN-F and RT-R (Additional file 4) resulted in 687 bp and 156 bp amplicons, respectively. The sequences of these amplicons contain a 7 nucleotide sequence that is unique to AAEL010714-RA (Additional file 4), supporting that only this target is amplified. The sequenced cDNAs revealed single nucleotide polymorphisms within the Higgs strain and also between Higgs and Liverpool-derived AAEL010714-RA sequences. Blastx analysis of the conceptual translation product of the 687 bp cDNA sequence using the Vectorbase databank showed the highest similarity with AAEL010714-PA followed by AAEL010718-PA.

### Temporal- and tissue-specific expression of AAEL010714

**Figure 1:**
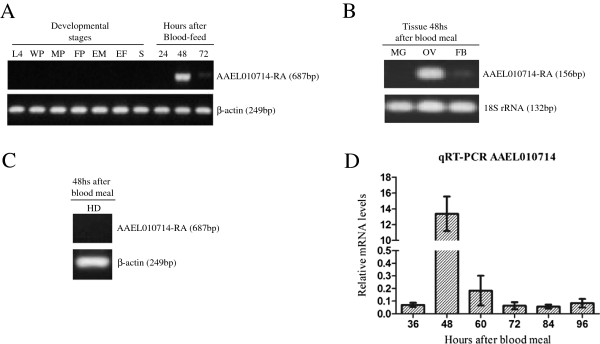
**Gene amplification analyses of transcription profiles of AAEL010714. A)** mRNA accumulation profiles in developmental stages and times after adult females feed on blood: fourth instar larvae (L4), white pupae (WP), male pupae (MP), female pupae (FM), newly-emerged males (EM), newly-emerged females (EF), sugar meal adult females (S), and adult females 24, 48 and 72 hPBM. β-actin mRNA levels were used as a control. **B)** Tissue-specific expression profile. All tissues, midgut (MG), ovaries (OV) and fat bodies (FB), were dissected from *Ae. aegypti* females at 48 hPBM. The same RNA samples were also analyzed by 18S rRNA-specific primers (Additional file 1 - [25]) as a control. **C)** Expression profile in female heads dissected at 48 hPBM. β-actin specific primers were used as controls on the same RNA samples. **D)** Quantification of accumulated of AAEL010714 transcripts by qRT-PCR in adult females 36, 48, 60, 72, 84 and 96 hPBM. All data represent mean values and error bars indicate standard error of the mean; *: p < 0.0001.

AAEL010714 transcripts are observed only in females and are accumulated highly at 48 hPBM (Figure 1A). The transcripts are most abundant in ovaries with comparatively lower levels in the fat body (Figure 1B). No transcripts are detected in female midguts. Despite the AEL010714 translation product being annotated as an OBP, no corresponding transcripts were detected in head samples (Figure 1C).

A one-way ANOVA statistical analysis performed with normalized values of transcript copy numbers confirmed that AAEL010714 transcripts are most abundant in vitellogenic females at 48 hPBM. The measured transcript levels differ significantly (p < 0.0001) between females at 48 hPBM and all other sample points (Figure 1D).

### Detection of AaegOBP45 in mosquito ovaries

**Figure 2:**
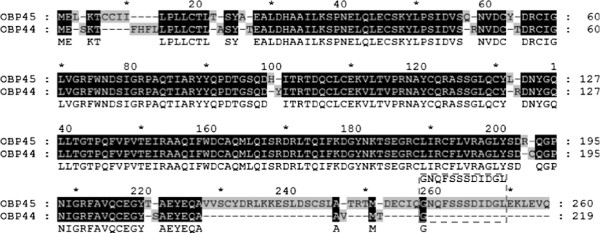
**The alignment of the predicted polypeptides encoding *Ae. aegypti *odorant-binding proteins OBP45 and OBP44.** Amino acids sequences were obtained from the Vectorbase databank and were annotated previously as AaegOBP45 and AaegOBP44 [14]. The numbers above the alignment indicate amino acid position in the whole alignment and the numbers on the right of the alignment show amino acid residue positions in each sequence. Conserved and non-conserved residues are shaded black or gray, respectively. A consensus sequence (non-shaded) is displayed on the third line. Dashes indicate gaps in sequences. Dashed gray box highlights the dodecapeptide sequence (GNQFSSSDIDGL) used to produce a synthetic peptide and polyclonal antibodies.

**Figure 3:**
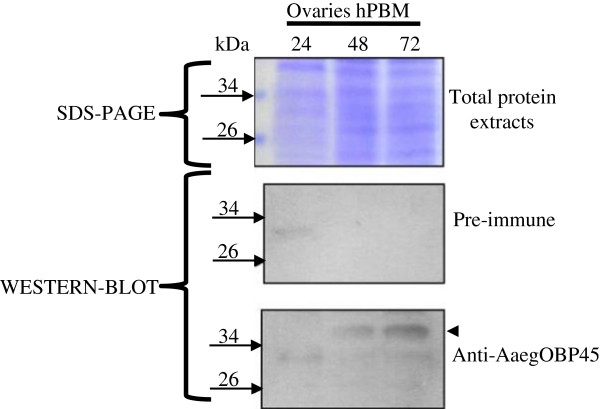
**Immunoblot of the total protein extracts from *Ae. aegypti *ovaries following a blood meal.** Top panel, coomassie stained gel of total protein extracts. The positions of marker proteins are indicated by arrows with the M_r_ of each listed. Middle panel, control immunoblot reacted only with the secondary antibody. Bottom panel, immunoblot reacted with both primary (anti- AaegOBP45) and secondary antibody sera. The arrowhead indicates the immunoreactive peptides.

Polyclonal antisera against the AaegOBP45-GNQFSSSDIDGL (Figure 2) peptide reacts with a protein present in 48 and 72 hPBM ovaries (Figure 3). The protein is not detected at 24 hPBM, consistent with the gene transcriptional profile (Figure 3). The predicted molecular mass of the mature AaegOBP45 is 26.8 kDa. However, the protein reacting with the antibodies displays a higher apparent molecular mass. Two potential sites for N-linked glycosylation (Asn^44^ and Asn^147^) exist in AaegOBP45; therefore, post-translational modifications may account for the difference between the predicted and experimentally-derived apparent mass.

## Conclusions

Only two publications to date report the expression profiles of atypical OBPs in Culicidae. These atypical OBPs in *An. gambiae* are transcribed in chemosensory organs, as well as other tissues in adult and immature aquatic stages [10]. Two of the atypical OBPs in *Ae. albopictus* are expressed both in preadult stages and in adult hemolymph, and a third is expressed exclusively in antennae and legs [8]. In addition to chemosensory functions, transport of hydrophobic ligands in the hemolymph has been proposed as a role for the atypical *Ae. albopictus* OBPs.

Although 15 atypical OBPs were annotated in the *Ae. aegypti* genome [14], their transcriptional profiles have not been studied in detail. Our work validated the expression profile previously described [17], and further defined the accumulation of AAEL010714 transcripts in vitellogenic ovaries. Also, we demonstrated that the protein product AaegOBP45 is accumulated in the ovaries leading us to propose a new possible function involved with the reproductive mechanisms responsible for oocyte maturation. It has been shown that seven *An. gambiae* odorant-binding proteins are associated with mosquito eggshell, and four of them show restricted expression 48 hPBM [18]. Therefore, although functional validation of the atypical/two-domain AaegOBP45 is required, we hypothesize its involvement with eggshell formation.

## Competing interests

The authors declare that they have no competing interests.

## Author’s contribution

ALCS and MLC conceived the study. ALCS performed the molecular experiments as RNA extractions, RT-PCR, qRT-PCR, SDS-PAGE and *Western blot* assays. The author conducted all other analyses of the work as alignments and the statistical analysis and drafted the manuscript. BBK performed DNA genomic amplifications and sequencing, and participated in the design of the study. MLC, OM and AAJ participated in study design and coordination and helped to draft the manuscript. All authors read and approved the final manuscript.

## Supplementary Material

Additional file 1 Primers used in PCR, RT-PCR and qRT-PCR experiments. Click here for file

Additional file 2 **Quantification of β-actin accumulated transcripts by qRT-PCR in adult females 36, 48, 60, 72, 84, 96 hPBM.** RNA was extracted from three independent experiments, except for 84 hPBM (two independent), and each time point were measured three times. mRNA copy numbers were determined by comparison with known concentrations of a standard plasmid. No significant differences (p > 0.05) were observed between all the samples analyzed. All data represent mean values and error bars indicate standard error of the mean. Click here for file

Additional file 3 **Nucleotide sequence of the AAEL010714 gene from *Ae. aegypti *** **generated by manual sequencing.** Pentamer nucleotides under-lined with dots are a consensus putative arthropod initiator cap-site and the bold under-lined nucleotide is the putative start of transcription (+1). Solid and dashed underlined sequences represent exons (1 and 2) and introns, respectively. The putative TATA sequence is boxed. One putative *hunchback*-binding site is in a dashed box and potential *BR-C* gene family zinc-finger binding-sites are double underlined (non-aligned lines) highlighting two overlapped motifs. Nucleotide numbers are presented to the right and position was defined based on the putative first transcribed nucleotide. Click here for file

Additional file 4 **Alignment of the transcript nucleotide sequences AAEL010714-RA and AAEL010718-RA from Vectorbase with nucleotide sequences obtained experimentally.** BioEdit was used to align the sequences. Regions where primers were designed are boxed in gray line. Primer F and Primer RT-R were used for cDNA amplification. Primer IN-F and RT-R were used for cDNA amplification in tissue RT-PCR and Real-time RT-PCR experiments. Black boxed gap indicates that 7 nucleotides are missing in AAEL010718-RA (718-RA) which are present in AAEL010714-RA (714-RA) and in sequences obtained by RT-PCR (cDNA_partial/1-687 and cDNA partial/1-156). Dots represent gaps. Polymorphisms between all sequences are shaded gray. Click here for file
