# Supplementary figures and images for: Expression and accumulation of the two-domain odorant-binding protein AaegOBP45 in the ovaries of blood-fed Aedes aegypti
Source: Parasit Vectors. 2013 Dec 24;6:364. doi: 10.1186/1756-3305-6-364 (PMC3892079; doi:10.1186/1756-3305-6-364)

## Slide 1
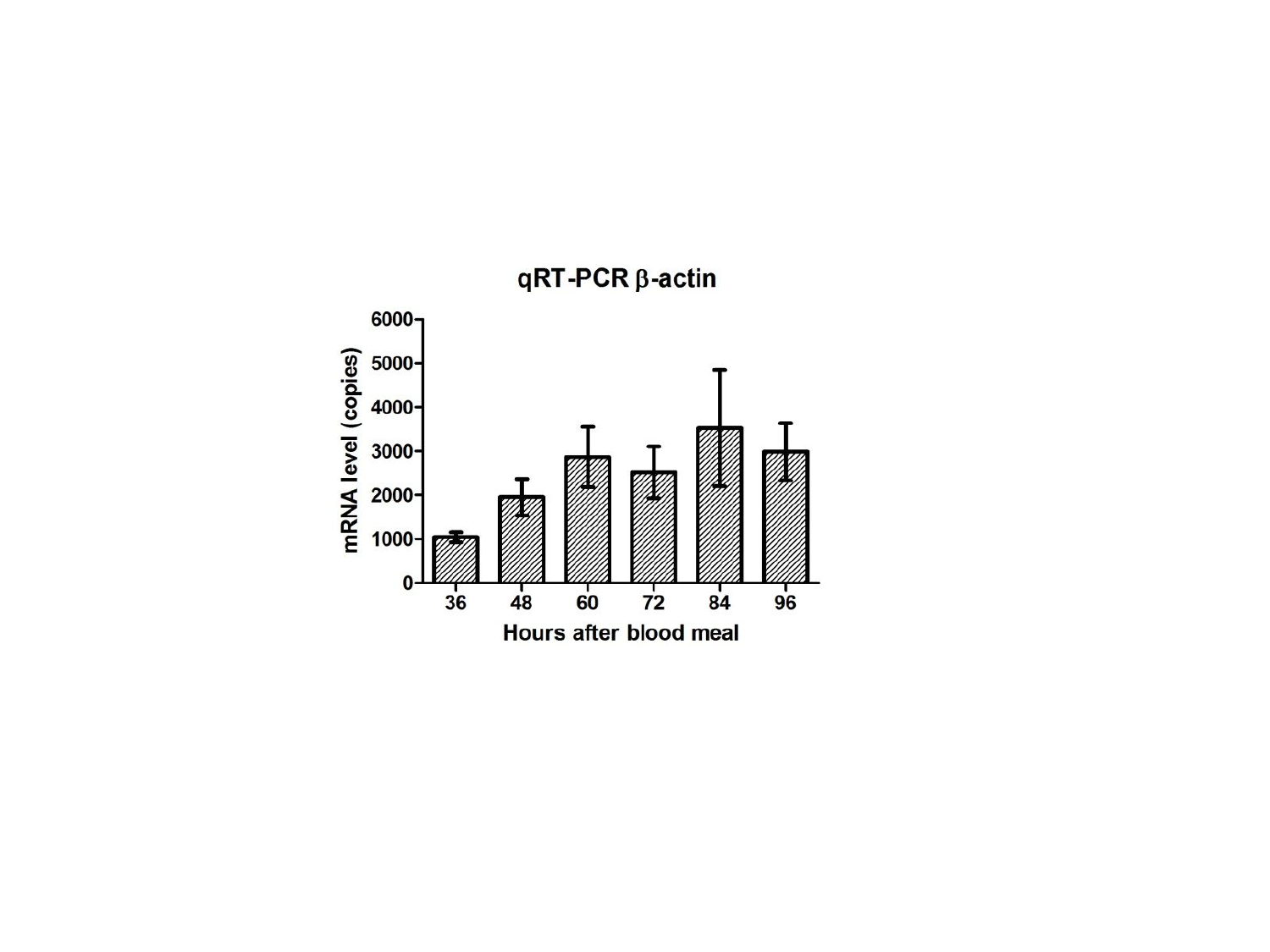

Supplement: Additional file 2 — Quantification of β-actin accumulated transcripts by qRT-PCR in adult females 36, 48, 60, 72, 84, 96 hPBM. RNA was extracted from three independent experiments, except for 84 hPBM (two independent), and each time point were measured three times. mRNA copy numbers were determined by comparison with known concentrations of a standard plasmid. No significant differences (p > 0.05) were observed between all the samples analyzed. All data represent mean values and error bars indicate standard error of the mean. [file 1756-3305-6-364-S2.pptx]

## Slide 1
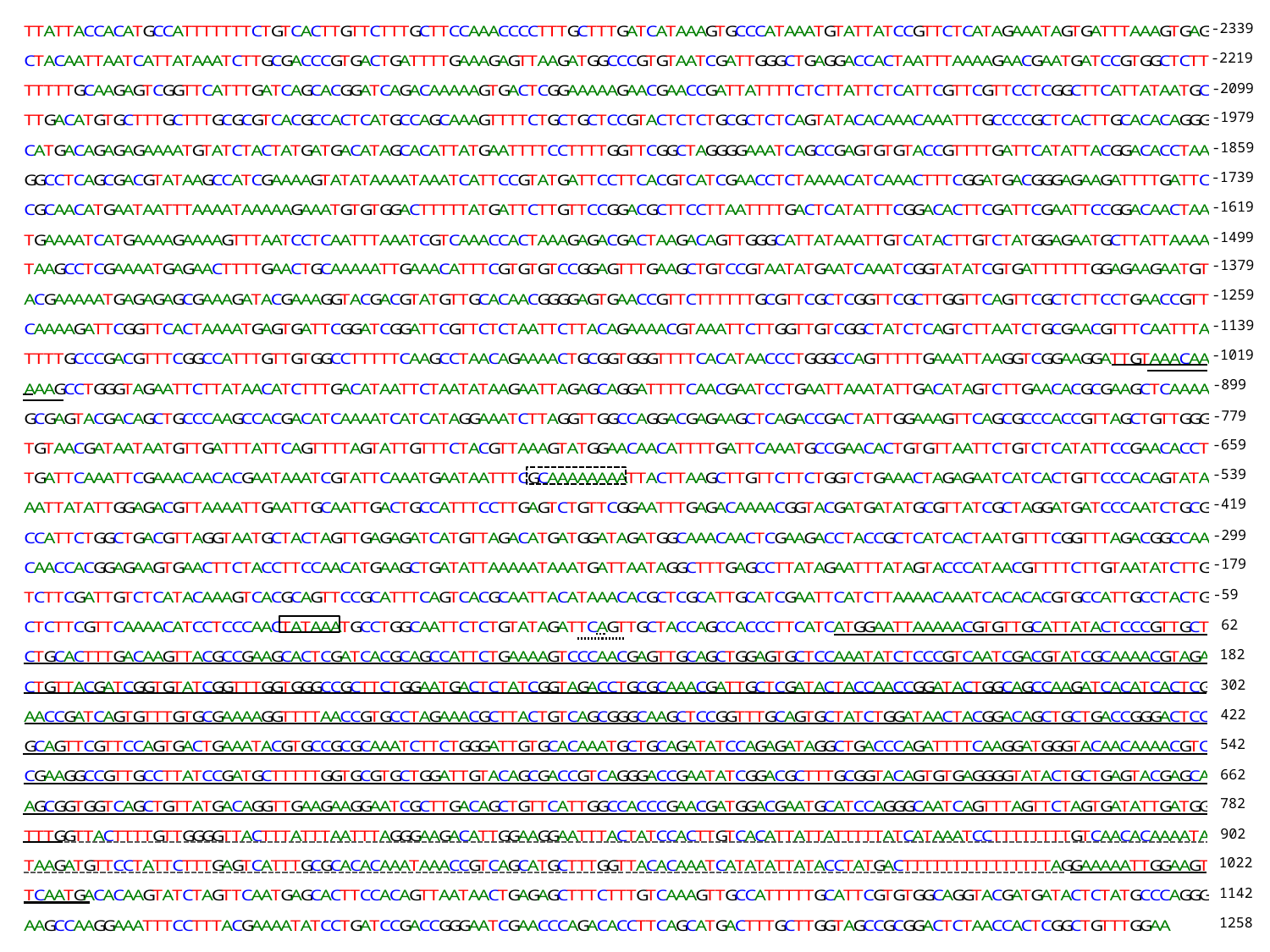

-2339
-2219
-2099
-1979
-1859
-1739
-1619
-1499
-1379
-1259
-1139
-1019
-899
-779
-659
-539
-419
-299
-179
-59
62
182
302
422
542
662
782
902
1022
1142
1258

Supplement: Additional file 3 — Nucleotide sequence of the AAEL010714 gene from Ae. aegypti generated by manual sequencing. Pentamer nucleotides under-lined with dots are a consensus putative arthropod initiator cap-site and the bold under-lined nucleotide is the putative start of transcription (+1). Solid and dashed underlined sequences represent exons (1 and 2) and introns, respectively. The putative TATA sequence is boxed. One putative hunchback-binding site is in a dashed box and potential BR-C gene family zinc-finger binding-sites are double underlined (non-aligned lines) highlighting two overlapped motifs. Nucleotide numbers are presented to the right and position was defined based on the putative first transcribed nucleotide. [file 1756-3305-6-364-S3.pptx]

## Slide 1
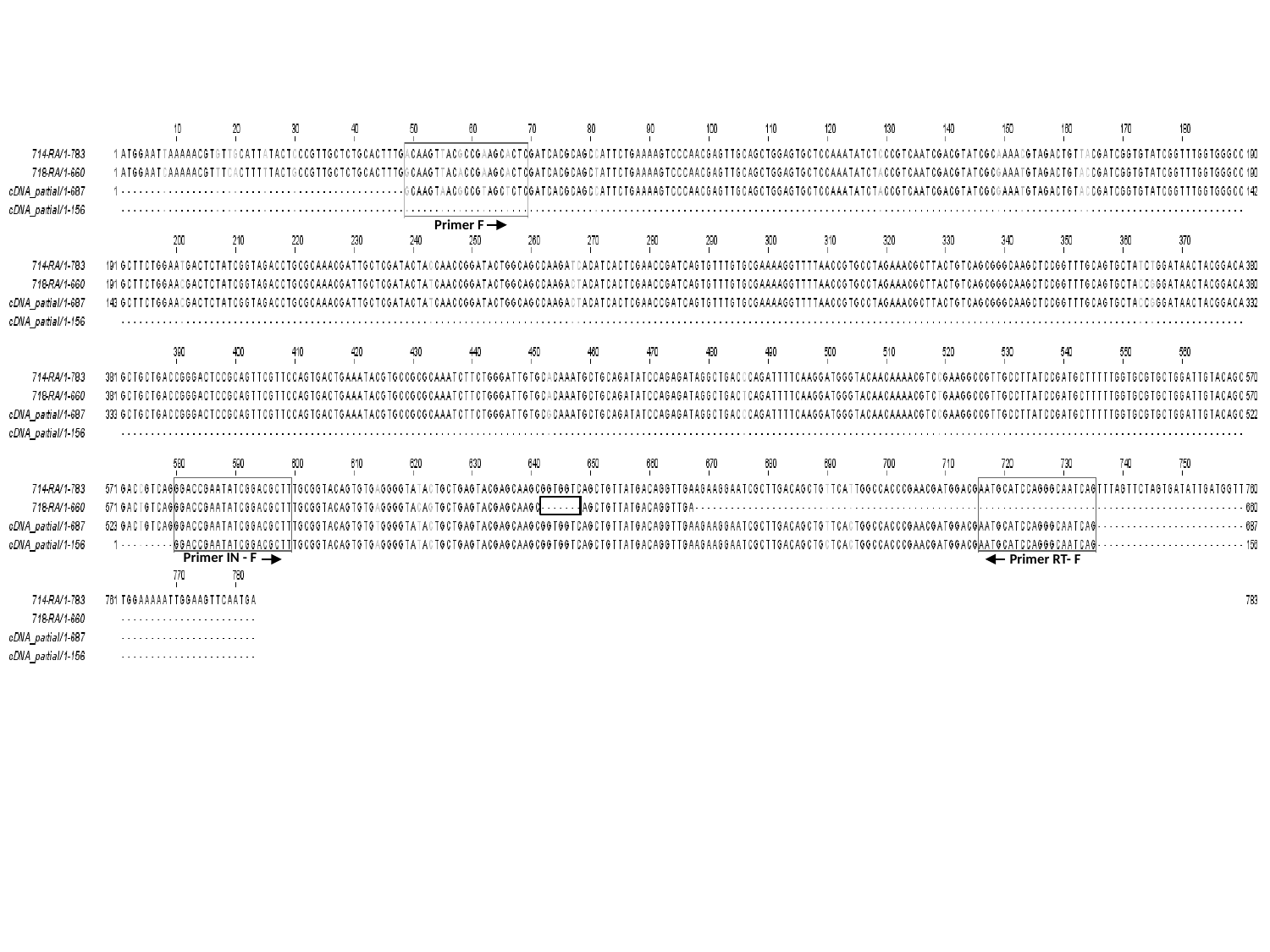

Primer F
Primer IN - F
Primer RT- F

Supplement: Additional file 4 — Alignment of the transcript nucleotide sequences AAEL010714-RA and AAEL010718-RA from Vectorbase with nucleotide sequences obtained experimentally. BioEdit was used to align the sequences. Regions where primers were designed are boxed in gray line. Primer F and Primer RT-R were used for cDNA amplification. Primer IN-F and RT-R were used for cDNA amplification in tissue RT-PCR and Real-time RT-PCR experiments. Black boxed gap indicates that 7 nucleotides are missing in AAEL010718-RA (718-RA) which are present in AAEL010714-RA (714-RA) and in sequences obtained by RT-PCR (cDNA_partial/1-687 and cDNA partial/1-156). Dots represent gaps. Polymorphisms between all sequences are shaded gray. [file 1756-3305-6-364-S4.pptx]
